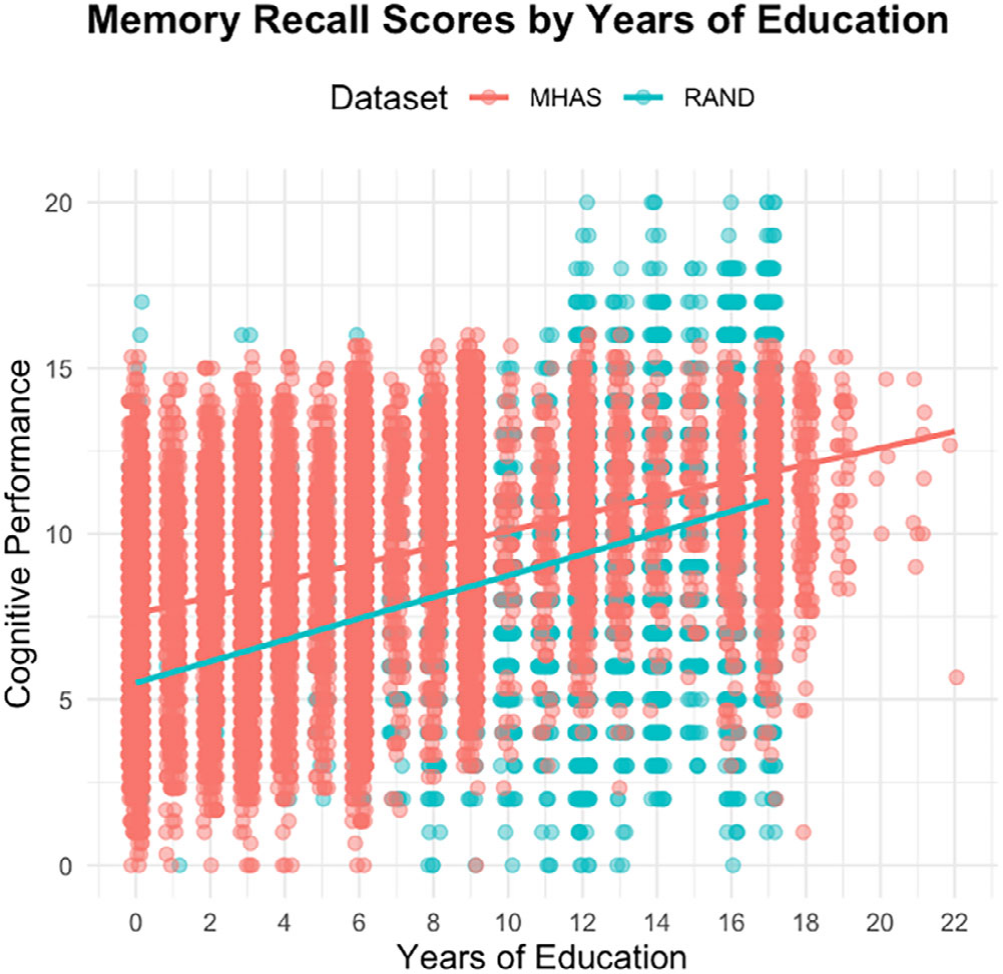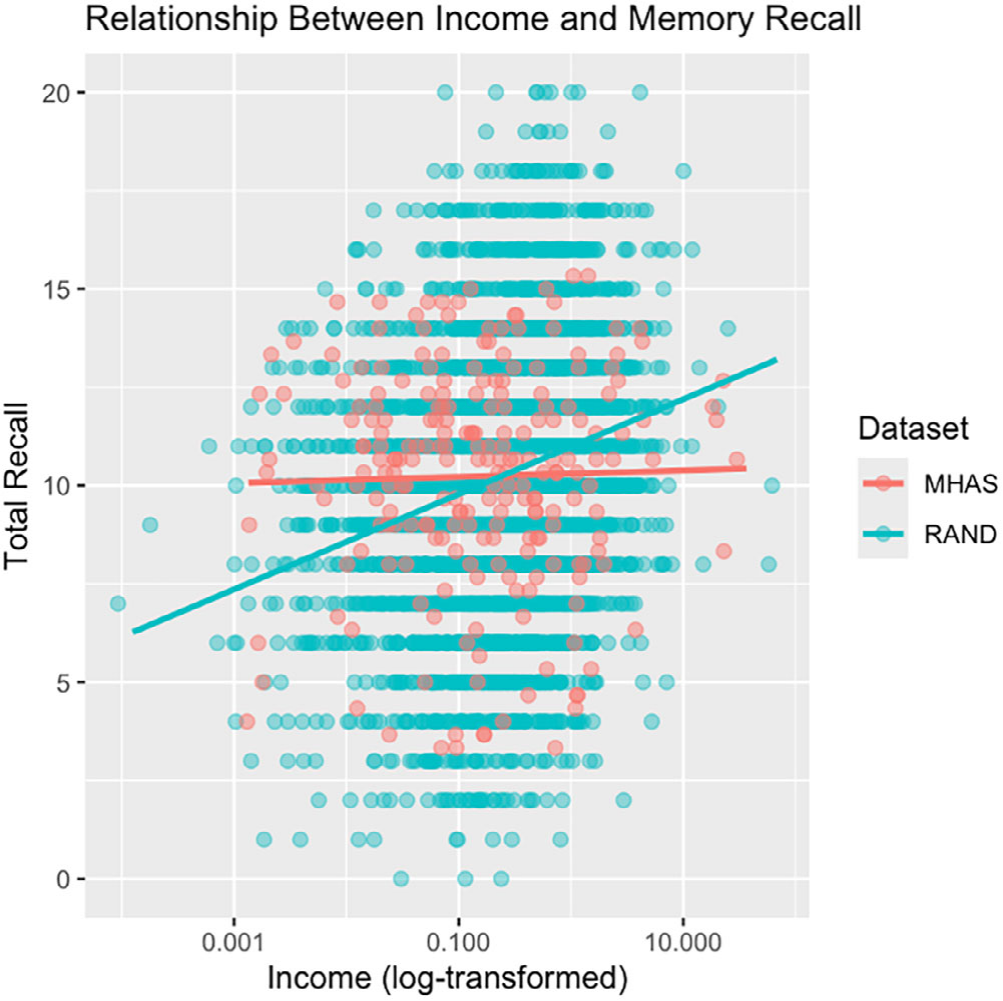# Social Determinants of Health and Cognitive Performance: A Cross‐National Study of the U.S. and Mexico

**DOI:** 10.1002/alz70858_106119

**Published:** 2025-12-24

**Authors:** Dalia Zizumbo, Karen S. Basurto, Nikole A Bonillas Félix, Whitney Cordoba, Moroni Fernandez Cajavilca, Alicia Goytizolo, Jairo E. Martinez, Tatiana Mercedes, Nathan Ramirez, Nadeshka J Ramirez‐Perez, Isabel Solis, Gladiliz Rivera‐Delpin, Glariangeliz Tapia‐Nazario, Averi Giudicessi, CADLAS Trainee Group

**Affiliations:** ^1^ Northeastern University, Boston, MA, USA; ^2^ Rosalind Franklin University of Medicine and Science, North Chicago, IL, USA; ^3^ Massachusetts General Hospital, Harvard Medical School, Boston, MA, USA; ^4^ Indiana University‐Bloomington, Bloomington, IN, USA; ^5^ New York University, New York, NY, USA; ^6^ Florida Atlantic University, Davie, FL, USA; ^7^ Boston University, Boston, MA, USA; ^8^ Ponce Health Sciences University, Ponce, PR, USA; ^9^ University of Pittsburgh Medical Center, Pittsburgh, PA, USA; ^10^ Brigham and Women's Hospital, Boston, MA, USA; ^11^ University of Puerto Rico, San Juan, PR, USA; ^12^ Cadlas Consortium, Boston, MA, USA

## Abstract

**Background:**

Social Determinants of Health (SDoH) such as socioeconomic status (SES), occupation, marital status, and healthcare access are modifiable risk factors associated with Alzheimer's disease and related dementias (ADRD). Though country‐specific factors influence SDoH, studies comparing their relationship with cognition between high and middle‐income countries are scarce. We leveraged harmonized datasets from the U.S. and Mexico to examine SDoH‐cognition relationships in community‐dwelling older adults.

**Method:**

Data included 7,075 Health and Retirement Study (HRS) and 12,893 Mexican Health and Aging Study (MHAS) participants. Cognitive measures included immediate recall, delayed recall, and serial 7s subtraction. The same cognitive tests were used between studies, adapted in Spanish for MHAS. We examined education, income, household size, marital status, and health insurance using multiple linear regression models with interaction terms between dataset and each social determinant, adjusting for age and sex. False discovery rate method controlled for multiple comparisons.

**Result:**

Compared to MHAS, HRS participants were older (M age=66.8 (SD=12.0) vs. 65.3 (12.0) years), and had more education (13.0 [SD=3.2] vs. 6.1 [SD=4.7]). After controlling for sociodemographic factors, HRS participants scored lower on all cognitive measures (β range: ‐0.605 to ‐2.25, all *p* <0.001). Education was the strongest predictor of cognitive performance for both MHAS and HRS (β: 0.429‐1.04, *p* <0.001). Income was positively associated with cognition (β: 0.068‐0.176, *p* <0.05), with stronger effects in HRS (β: 0.371‐0.797, *p* <0.001). Having government health insurance vs those without predicted better immediate recall in MHAS (β=0.110, *p* <0.01), and improved delayed recall in HRS (β=0.274, *p* <0.01). In HRS, larger households predicted lower recall scores (β: ‐0.180 to ‐0.230, *p* <0.001). Marriage was associated with better delayed and total recall performance in HRS (β: 0.362‐0.493, *p* <0.001).

**Conclusion:**

Findings highlight the complex influence of SDoH on cognitive performance. Despite higher education and income, HRS participants scored lower across cognitive measures. Education was the strongest predictor of cognitive performance in both countries, reinforcing its role as a key factor for promoting healthy cognitive aging. Results emphasize the importance of considering sociocultural contexts when developing cognitive health interventions.